# Weather data sources influence predictive performance of disease forecasting models

**DOI:** 10.1007/s00484-026-03285-3

**Published:** 2026-08-18

**Authors:** Theophilus Abonyi Mensah, Neil White, Vivian Rincón-Flórez, Olufemi A. Akinsanmi

**Affiliations:** 1 Centre for Horticultural Science, Queensland Alliance for Agriculture and Food Innovation, The University of Queensland, Ecosciences Precinct, Brisbane, QLD 4102 Australia; 2 Queensland Department of Primary Industries, Toowoomba, QLD 4350 Australia

**Keywords:** Airborne conidia, Disease forecasting, Flower blight, Microclimate, Weather-based models

## Abstract

Weather data from fine- and coarse-scale sources are increasingly used in disease forecasting models to support management decisions, yet comparisons between fine- and coarse-scale sources remain limited, largely due to the difficulty of acquiring fine-scale datasets across locations and seasons. Differences among weather sources can influence model outputs, resulting in variation in predictive performance and the timing of management decisions. This study evaluated four weather data sources, grouped as fine-scale (in-canopy and on-farm stations) and coarse-scale (regional gridded and point-based data from SILO, and Bureau of Meteorology, BOM, stations), across two production regions in Australia. Macadamia orchards were used as a case study to assess spatial variability in 10 weather variables associated with fungal infection over three seasons (2022–2024). Heterogeneous trends and associations were identified among the weather sources, primarily driven by differences in minimum temperature and minimum relative humidity. On-farm and SILO data showed high spatial variability, whereas in-canopy and BOM data were comparatively stable. Predictive performance of disease models based on each weather source was evaluated using airborne conidia dynamics for the fungal pathogens *Botrytis*, *Cladosporium* and *Neopestalotiopsis*/*Pestalotiopsis*, which cause flower blight in macadamia. In-canopy sources produced significantly lower predictive performance (*p* < 0.05) than BOM sources, while on-farm, SILO and BOM sources showed comparable performance (*p* > 0.05) across all pathogens. These findings indicate that the choice of weather data source, rather than spatial scale alone, is critical for reliable disease forecasting.

**Supplementary Information:**

The online version contains supplementary material available at https://doi.org/10.1007/s00484-026-03285-3.

## Introduction

Disease forecasting models offer a valuable tool to optimise management strategies and reduce fungicide wastage (Perria et al. 2022; Shtienberg 2013). Models may predict infection risks and disease epidemics for specific crops and regions, providing early warning signals that enable targeted fungicide applications based on actual disease risk rather than calendar schedules. In potatoes, the application of models such as TOMCAST and other infection-based forecasting systems are used for managing potato early blight (*Alternaria* species), which have reduced fungicide application frequency and quantity by more than 20%, while increasing profit margin by up to 9% compared with a fixed-schedule program (Abuley et al. 2023). Several predictive models have also been developed for timing infection and controlling diseases caused by *Diaporthe* (Phomopsis cane and leaf spot of grapevine), *Botryosphaeria* (larch shoot blight), *Botrytis* (grapevine bunch rot) and *Cladosporium* (tomato powdery mildew) (Gonzalez-Dominguez et al. 2022; Jensen et al. 2025; Zhao et al. 2024; Zhou et al. 2024). Accurate forecasting depends on selecting suitable predictor variables, and includes knowledge of the pathogen life cycle, host phenology and environmental variables required for infection and disease development (Jensen et al. 2025).

Weather variables are the most commonly used predictors of disease epidemiology because they directly influence pathogen development and infection processes and are relatively easy to obtain (Jensen et al. 2025). Air temperature, dew point temperature, relative humidity, rainfall and leaf wetness data have been associated with the progression of several crop diseases (Rodríguez-Moreno et al. 2020). A generic forecasting framework for various pathosystems was developed by scaling leaf wetness duration to a temperature response function involving maximum, minimum, mean and optimum temperatures for pathogen infection (Magarey et al. 2005). In macadamia, relative humidity and rainfall have been identified as major weather variables associated with dynamics of Phomopsis husk rot and Botryosphaeria branch dieback pathogens, respectively (Mohankumar et al. 2023; Prasannath et al. 2022a). Preliminary regression analyses suggest that these variables could potentially explain variation in airborne conidia abundance for both pathosystems (Mohankumar et al. 2023; Prasannath et al. 2022a).

The source and quality of weather data used as inputs in disease models may influence predictive accuracy. Weather data are generally obtained from two spatial scales: farm-level (fine-scale) and regional-level (coarse-scale) sources. Fine-scale weather sources include in-canopy stations, with sensors that capture microclimatic conditions located within the crop canopy, and on-farm stations, located in open areas outside the canopy. Fine-scale sources are often expensive to install and maintain, and their placement may disrupt farm operations. Their accuracy may also be compromised by improper sensor installation or operator inexperience (Gleason et al. 2008). In contrast, coarse-scale data from government-maintained networks such as the Australia Bureau of Meteorology (BOM) and the Scientific Information for Land Owners (SILO) are more accessible and widely used for large-scale modelling (Brinkhoff and Robson 2021). BOM provides point-based measurements with a spatial resolution of up to 60 km, whereas SILO offers point-based and 5 km gridded datasets derived from BOM, providing finer coverage (Wedd et al. 2022; Zaman et al. 2024). Assessing predictive models, Brinkhoff and Robson (2021) noted that predictions from finer scale models tend to be more accurate when evaluated at coarser scales. However, Deines et al. (2021) emphasised that model accuracy is scale-dependent. To identify the most informative and reliable weather data source for disease forecasting tools applicable across regions, it is essential to assess how potential variability between fine- and coarse-scale data affects the predictive performance of disease models. In this study, we used macadamia as a case study.

Macadamia is a high value commercial tree nut crop cultivated in tropical and subtropical regions worldwide. China (32%), South Africa (28%), Australia (13%), Kenya (10%) and the United States (4%) accounts for over 82,000 metric tonnes (kernel basis) with supply value of more than $640 million (INC 2025). Despite increasing production trends, macadamia cultivation is challenged by fungal diseases that cause significant yield losses, including flower blight, husk rot, husk spot and Botryosphaeria branch dieback (Akinsanmi and Drenth 2017; Akinsanmi et al. 2007; Mohankumar et al. 2023; Prasannath et al. 2023). These diseases develop across nearly all phenological stages and are mostly managed using integrated approaches, including fungicide application. In macadamia, phenological development begins with budbreak in early winter, followed by raceme elongation in mid-winter and flowering from mid-winter to early spring (Bright 2025). Flowering comprises four distinct stages: punching bag (stage 1), style emergence (stage 2), peak anthesis (stage 3) and swollen ovary (stage 4). The swollen ovary develops into the matchhead and pea-size nut set stages during spring. Fruit development then progresses to the full-size nut stage, characterised by shell hardening and oil accumulation, before reaching physiological maturity in summer. Previous studies have established the critical role of weather conditions in disease development across these phenological stages (Akinsanmi and Drenth 2010, 2017; Mohankumar et al. 2023).

Flower blight is the most frequent and economically significant disease affecting flowers and can severely reduce pollination efficiency, causing over 99% fruit set failure and 80% yield loss (Prasannath et al. 2021, 2022a). Flower blight is caused by a complex of fungal pathogens of contrasting lifecycles, including several species of *Pestalotiopsis*, *Neopestalotiopsis*, *Botrytis* and *Cladosporium*, and risk periods (Prasannath et al. 2023). These fungal pathogens produce abundant airborne conidia that are primarily dispersed by wind. The peak risk periods are from budbreak to swollen ovary for *Pestalotiopsis* and *Neopestalotiopsis*, elongated raceme to swollen ovary for *Cladosporium* and punching bag to peak anthesis for *Botrytis*. Disease onset and severity are strongly influenced by weather conditions, including leaf wetness, temperature, rainfall and vapour pressure deficit (Molitor et al. 2020; Navi et al. 2005; Prasannath et al. 2022a). Hence, improved knowledge on the specific timing and duration of infection risk is crucial for effective disease control, making forecasting a useful early diagnostic tool.

The quantitative nature of fine- and coarse-scale weather data inputs in disease forecasting remain poorly understood, limiting their harmonisation for practical on-farm application. Weather data from fields within 15 km can result in different infection risk predictions (El Jarroudi et al. 2017; Rodríguez-Moreno et al. 2020). To evaluate the predictive performance of fine- and coarse-scale weather data for disease forecasting in macadamia orchards, we hypothesised that fine-scale sources provide greater predictive accuracy than coarse-scale sources. Specifically, this study aimed to (i) explore the relationship between weather variables from fine-scale and coarse-scale sources, (ii) assess the predictive performance of VPD-based disease models across different growing regions and (iii) determine the impact of weather data source on predicting fungal flower blight in macadamia.

## Materials and methods

### Sources of weather data from case study sites

**Fig. 1:**
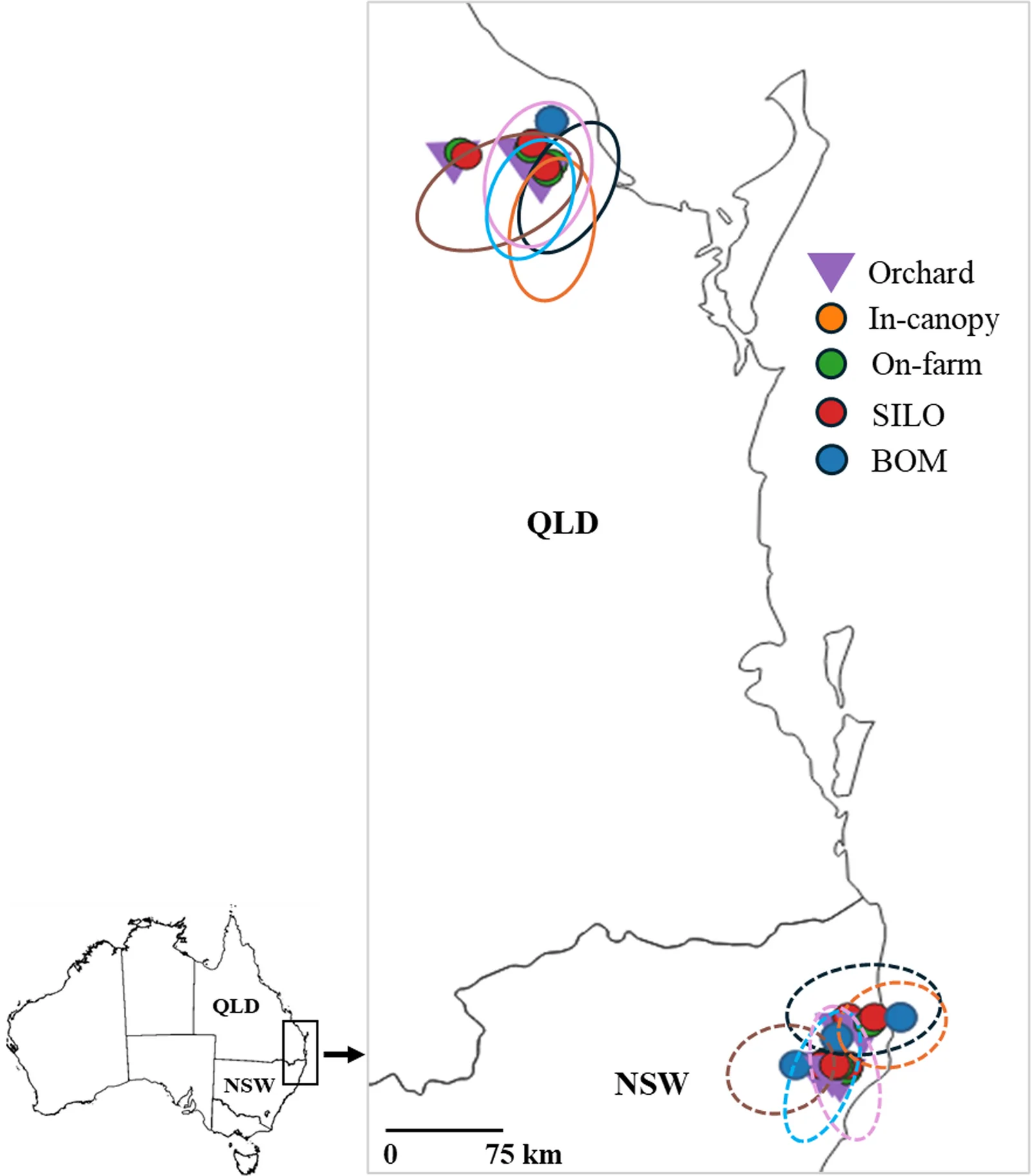
Location of orchards and their corresponding weather sources used in the study. Each circle indicates an orchard and its associated weather sources in Central Queensland (solid circles): Orchard 1 (black), Orchard 2 (orange), Orchard 3 (brown), Orchard 7 (blue) and Orchard 8 (pink); and Northern Rivers, New South Wales (dashed circles): Orchard 4 (black), Orchard 5 (orange), Orchard 6 (brown), Orchard 9 (blue) and Orchard 10 (pink)

The case study sites included 10 commercial macadamia orchards in two agroecologically distinct regions, with five orchards each in the Central Queensland (CQ) and Northern Rivers (NR), New South Wales in Australia (Fig. 1). The regions and orchards were selected based on the availability of fine-scale weather sources and records (up to 3 years). A summary of the orchard characteristics is shown in Table 1. The tree ages ranged from 4 to 22 years, with mean canopy diameters of about 5 m in CQ and 8 m in NR (Table 1). For each orchard, weather data were collected from two fine-scale and two coarse-scale sources. The fine-scale sources included in-canopy and on-farm stations. Data from both fine-scale sources were accessed from Davis weather stations through the WeatherLink platform (https://www.weatherlink.com/). The coarse-scale sources comprised BOM (http://www.bom.gov.au/climate/data) and SILO (www.longpaddock.qld.gov.au/silo/point-data/). BOM and SILO provide regional data and were regarded as coarse-scale based on their distances from the orchards (Table 1), calculated using the Haversine formula (Basyir et al. 2017) as

1 $$\begin{array}{c}\:d_s=2r\:arcsin\\\sqrt{{sin}^2\left(\frac{{\phi\:}_2-{\phi\:}_1}2\right)+cos\left({\phi\:}_1\right).cos\left({\phi\:}_2\right).{sin}^2\left(\frac{{\psi\:}_2-{\psi\:}_1}2\right)}\end{array}$$

**Table 1:** Characteristics and distances (km) of selected commercial macadamia orchards to nearest coarse-scale weather data sources in Central Queensland (CQ) and Northern Rivers (NR), with corresponding number and range of observation years

Region	Orchard characteristics			Distance to coarse-scale sources (km)		Range of observation years
	Label	Tree age (years)	Mean canopy diameter (m)	SILO	BOM (station ID)	
CQ	Orchard 1	8	5.96	2.94	19.87 (39128)	2022–2024
	Orchard 2	4	2.44	2.31	23.68 (39128)	2022–2024
	Orchard 3	5	5.30	2.82	37.91 (39128)	2022–2024
NR	Orchard 4	20	10.32	2.63	19.22 (58216)	2022–2024
	Orchard 5	22	9.21	3.05	11.75 (58216)	2022–2024
	Orchard 6	13	8.47	2.11	11.37 (58214)	2022–2024
CQ	Orchard 7	8	5.87	2.58	15.27 (39128)	2023–2024
	Orchard 8	17	6.33	2.47	15.09 (39128)	2023–2024
NR	Orchard 9	5	4.95	2.57	15.71 (58198)	2024
	Orchard 10	15	8.03	3.61	13.00 (58198)	2024

In-canopy and on-farm stations were located within the orchards (distance to orchard = 0 km). BOM: Bureau of Meteorology (Australian Government); SILO: Scientific Information for Land Owners (Queensland Government)

where $\:{d}_{s}$ is the distance (km), *r* is the radius of the earth (6371 km), and $\:{\phi\:}_{1}$, $\:{\psi\:}_{1}$ and $\:{\phi\:}_{2}$, $\:{\psi\:}_{2}$ are the latitudes and longitudes (radians) of an orchard and coarse-scale source.

### Weather variables

Weather data from July (winter) to December (summer) in the 2022 to 2024 production seasons were used (Table 1). This period coincides with the flowering and fruit development stages for macadamia in Australia. Weather variables common across the four sources were selected, including maximum temperature ($T_{max}$, °C), minimum temperature ($T_{min}$, °C), maximum relative humidity ($RH_{max}$, %), minimum relative humidity ($RH_{min}$, %) and rainfall (mm). Rainfall data were not available for the in-canopy stations. Therefore, in-canopy stations were excluded from rainfall-related analyses. Five weather variables commonly used in disease models were derived for each weather source, including VPD (kPa), leaf wetness duration (LW, days), maximum dew point temperature ($T_{dmax}$, °C), minimum dew point temperature ($T_{dmin}$, °C) and thermal oscillation ($T_{o}$, °C) (Abuley et al. 2023; Manstretta and Rossi 2015; Sadyś et al. 2018; Vélez-Pereira et al. 2019). $T_{o}$ was calculated as the difference between $T_{max}$ and $T_{min}$ while LW was calculated using the fixed RH threshold approach, with a day considered wet if RH ≥ 90% (Sentelhas et al. 2008). The fixed RH threshold provides an indirect estimate of LW and may not fully capture the actual LW measurements taken using sensors (Rowlandson et al. 2015). VPD, $T_{dmax}$ and $T_{dmin}$ were calculated (Allen et al. 1998; Lawrence 2005) as

2 $$\begin{array}{c}VPD=\left(\frac{A_1exp\left[\frac{B_1T_{max}}{T_{max}+C}\right]+A_1exp\left[\frac{B_1T_{min}}{T_{min}+C}\right]}2\right)-\\\:\left(\frac{A_1exp\left[\frac{B_1T_{max}}{T_{max}+C}\right]\left[\frac{{RH}_{min}}{100}\right]+A_1exp\left[\frac{B_1T_{min}}{T_{min}+C}\right]\left[\frac{{RH}_{max}}{100}\right]}2\right)\end{array}$$

3 $$\:T_{dmax}=\frac{B_2\left[ln\left(\frac{{RH}_{min}}{100}\right)+\frac{A_2T_{max}}{B_2+T_{max}}\right]}{\begin{array}{c}A_2-ln\left(\frac{{RH}_{min}}{100}\right)-\frac{A_2T_{max}}{B_2+T_{max}}\\\:\:\end{array}}$$

4 $$\:T_{dmin}=\frac{B_2\left[ln\left(\frac{{RH}_{max}}{100}\right)+\frac{A_2T_{min}}{B_2+T_{min}}\right]}{\begin{array}{c}A_2-ln\left(\frac{{RH}_{max}}{100}\right)-\frac{A_2T_{min}}{B_2+T_{min}}\\\:\:\end{array}}$$

where * exp* is exponential function, * ln* is logarithmic function and $\:{A}_{1}$ = 0.6108, $\:{B}_{1}$ = 17.27, * C* = 237.3, $\:{A}_{2}$ = 17.625 and $\:{B}_{2}$ = 243.04 are constants.

Coarse-scale sources (BOM and SILO) recorded daily data, while fine-scale sources (in-canopy and on-farm) recorded data at 5–60 min intervals depending on the orchard. Fine-scale data were standardised to daily resolution using the same procedure for coarse-scale data (BOM 2004). Some missing and aberrant records were observed in fine-scale sources and were excluded from the analyses.

All analyses and graphical visualisations were performed in Python version 3.12.7 using the packages; pandas (McKinney 2010), numpy (Harris et al. 2020), matplotlib (Hunter 2007), seaborn (Waskom 2021), pymannkendall (Hussain and Mahmud 2019), scipy (Virtanen et al. 2020), statsmodels (Seabold and Perktold 2010), scikit-learn (Pedregosa et al. 2011) and pca (Taskesen 2020).

#### Comparison of weather variables from fine- and coarse-scale sources

To assess variability in weather variables, six orchards, three in CQ (Orchards 1–3) and three in NR (Orchards 4–6) were used. These orchards had data in all sources for the 2022–2024 periods. Data were expressed as 14-day moving averages to visualise temporal trends. Short-term temporal dependence was quantified using lag-1 serial correlations ($\:{r}_{1}$) (Seabold and Perktold 2010). Serially correlated variables were pre-whitened using the trend-free pre-whitening (TFPW) procedure (Yue et al. 2002) to generate serially independent data for trend analysis. Trend magnitude was estimated using Theil-Sen’s nonparametric method (Sen 1968; Theil 1992). All tests were performed at a 5% significance level. Monotonic trends and directional changes at four phenological stages were then quantified using the standardised Mann-Kendall statistic ($Z_{mk}$) (Kendall 1948; Mann 1945).

To examine inter-source similarity, bivariate (pairwise correlations) and multivariate (clustering) approaches were used. Pairwise correlations were computed using Spearman’s rank correlation coefficient (*ρ*) to assess direct variable-to-variable agreement between sources, as the serially independent datasets significantly deviated from normality (Shapiro-Wilk test, *p* < 0.05) (Shapiro and Wilk 1965; Spearman 1904). Component-based clustering was performed on principal component scores using *k*-means and Gaussian mixture model to capture overall source-level variability (Ssegane et al. 2012). Silhouette analysis suggested varied optimal cluster numbers across orchards; therefore, a fixed number of four clusters, corresponding to the number of weather sources per orchard, was used. Clustering performance was evaluated using classification metrics, including confusion matrix, precision, recall, F1-score and overall accuracy. Precision is the proportion of correctly assigned sources within a cluster, recall is the proportion of true sources correctly assigned and F1-score is their harmonic mean. Accuracy reflects the proportion of correctly classified sources across all clusters. All metrics range from 0 to 1, with 1 indicating optimal performance (Vujović 2021).

#### Predictive performance of weather variables for flower blight pathogens

The predictive performance of source-specific weather variables was assessed by comparing predicted airborne flower blight conidial abundance to field observations from orchard traps. Data from four orchards in the 2023 and 2024 seasons were used. In CQ, Orchards 7 and 8, and in NR, Orchards 9 and 10 were selected because of available spore trapping systems. Airborne conidia of flower blight pathogens (*Botrytis*, *Cladosporium* and *Pestalotiopsis*/*Neopestalotiopsis*) were trapped using passive spore samplers constructed from rectangular plastic bottles assembled into opposing L-shaped sections (15.0 cm × 5.5 cm). Vaseline-coated Melinex tape (10.0 cm × 1.9 cm) was affixed to the internal surfaces. Traps were installed on branches at 1.8 m above the orchard floor to ensure uniformity (González-Fernández et al. 2020). Melinex tapes were collected bi-weekly from flowering (winter) to shell hardening and oil accumulation (summer). The number of conidia was quantified using multiplex quantitative PCR (qPCR) assay as described by Prasannath et al. (2022b).

Multivariate regression models were developed to relate conidial abundance of each pathogen during the spore-trapping period for each weather source. However, different predictor variables were identified as optimal among the weather data sources. For direct comparison of predictive performance across the weather sources, univariate regression models were developed using VPD from each weather data source. VPD was selected to standardise comparisons among weather sources rather than to maximise prediction accuracy, based on the previously reported polynomial association between flower blight conidial abundance and VPD (Prasannath et al. 2022a). Different temporal VPD structures were identified among the weather data sources. Hence, contemporaneous mean VPD from each weather source was used as the standardised predictor. Polynomial (p0 = 0.5, 1; maxfev = 10000) and simple linear regression models were evaluated, and the best-performing model type across weather sources was selected based on their predictive performance. Model development and evaluation were performed using the leave-one-out cross-validation (LOO-CV) method (De Cól et al. 2024), where each observation was iteratively held-out as the validation sample while the remaining observations were used as the training set (Hastie et al. 2009; Maleki et al. 2020). Predicted and observed conidial abundance from all iterations were aggregated to compute overall performance metrics, including the coefficient of determination ($\:{R}^{2}$), Lin’s concordance correlation coefficient (LCCC), coefficient of residual mass (CRM) and normalised root mean square error (NRMSE), describing variance explained, agreement, bias and error (Ibrahim et al. 2022; Jadon et al. 2024; Lin 1989). For NRMSE, the observed conidia range was used as the normalising factor.

Performance metrics were min-max normalised to an identical scale (0–1) to ensure equal weighting among metrics and minimise scale-related bias during comparison of predictive performance among the weather sources across orchards. Predictive performance among weather sources was compared using the Wilcoxon signed rank (*W*) and Kruskal-Wallis (*H*) tests (Kruskal and Wallis 1952; Wilcoxon 1945). Pairwise comparisons were performed using Dunn’s post hoc test with Bonferroni correction (Dunn 1964). To identify the best-performing weather source, a multi-criteria prediction robustness index (PRI) was calculated as

5 $$\:PRI=\overline N-(\lambda\:\times\:{SD}_n)$$

where $\:\overline{N}$ is the mean of the normalised performance metrics representing predictive performance, and $\:{SD}_{n}$ is the standard deviation of the normalised metrics across orchards representing performance variability. The weighting parameter ($\:\lambda\:$) functions as a penalty term to control the trade-off between maximising predictive performance and minimising variability in performance across orchards. Subtracting ($\:\lambda\:\times\:{SD}_{n}$) from $\:\overline{N}$ penalises weather sources that showed high cross-orchard variability to reduce potential effects of isolated orchard-specific performance extremes on overall PRI rankings. To select the $\:\lambda\:$ value, a sensitivity analysis was performed using $\:\lambda\:$ values ranging from 0 to 1. The resulting PRI values were compared across weather sources for the tested $\:\lambda\:$ range. A final $\:\lambda\:$ value (0.1), corresponding to the minimal penalty, was selected for the robustness analysis.

## Results

### Variability in weather variables based on sources

**Fig. 2:**
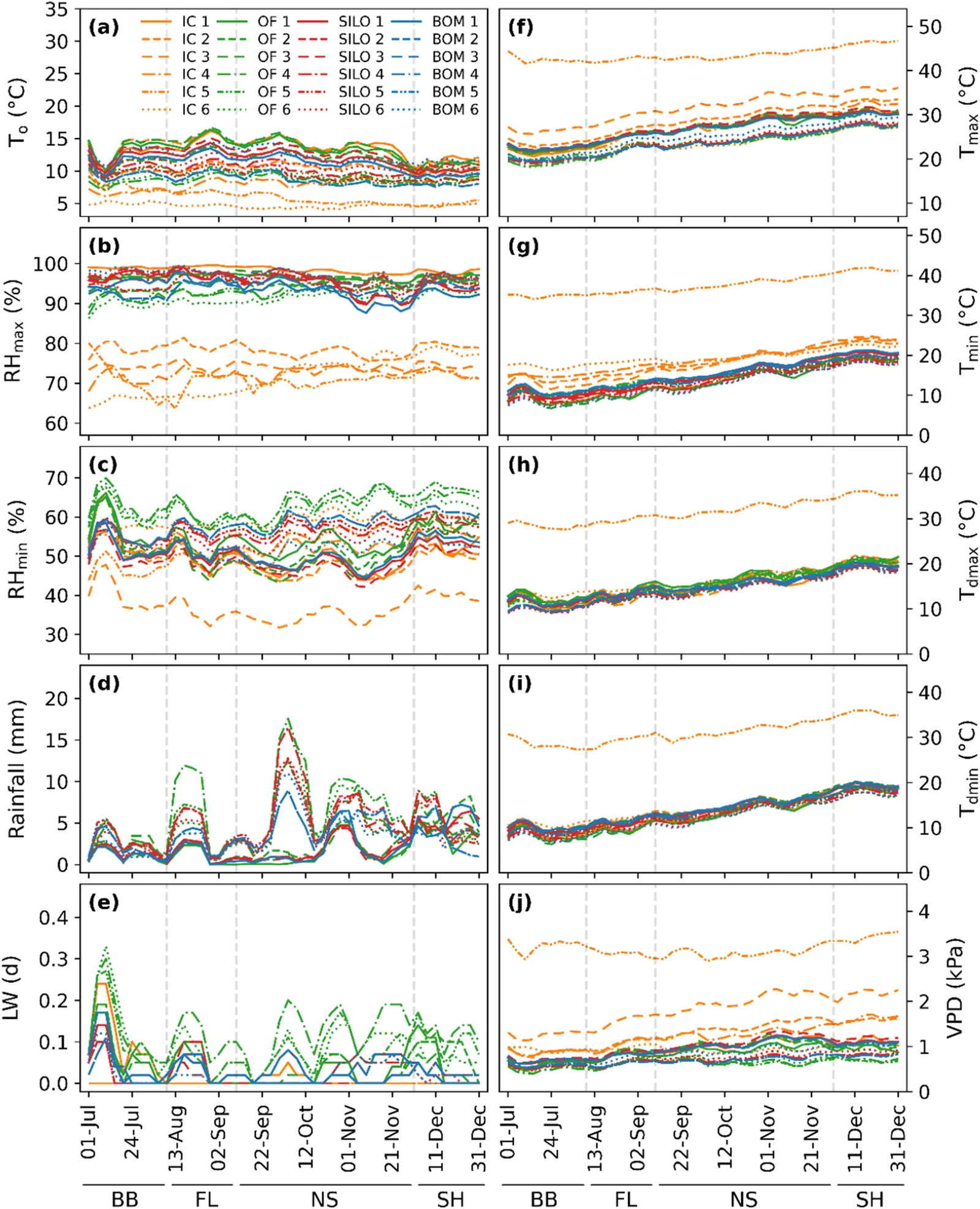
Moving averages (14-day) of 10 weather variables from fine-scale (in-canopy, IC and on-farm, OF) and coarse-scale (SILO and BOM) sources for six commercial macadamia orchards in Central Queensland (Orchards 1–3) and Northern Rivers (Orchards 4–6) across four developmental stages (BB: budbreak, FL: flowering, NS: Nut set and spring flush, and SH: Shell hardening and oil accumulation) in 2022 to 2024, with starting day set to 1 July for each season. $\:T_{o}$: thermal oscillation; $\:RH_{max}$: maximum relative humidity; $\:RH_{min}$: minimum relative humidity; LW: leaf wetness; $\:T_{max}$: maximum temperature; $\:T_{min}$: minimum temperature; $\:T_{dmax}$: maximum dew point temperature; $\:T_{dmin}$: minimum dew point temperature; VPD: vapour pressure deficit. Rainfall was not applicable for in-canopy stations. Numbers in the legend correspond to orchard labels

Temporal analysis revealed similar trends in most of the weather variables across the four sources (Fig. 2). Serial dependence between consecutive daily values of variables was detected across all sources, except for BOM, where rainfall (Orchards 1–3) and LW (Orchards 4–5) were largely independent (Table S1). A more stable microclimate was observed at in-canopy sources (mean $\:{r}_{1}$ = 0.74) compared with on-farm (mean $\:{r}_{1}$ = 0.71), SILO (mean $\:{r}_{1}$ = 0.71) and BOM (mean $\:{r}_{1}$ = 0.69) sources. A relative increase in $T_{max}$, $T_{min}$, $T_{dmax}$, $T_{dmin}$ and VPD ($\:{Z}_{mk}$ = 2.02–4.64, *p* < 0.05) with similar magnitudes (mean slope = 0.01) was observed beginning from the budbreak (Fig. 2f–j, Fig. S1, Table S2). In contrast, $RH_{max}$, $RH_{mi}$ and $T_{o}$ were more source-specific, with orchard-specific increases, decreases or no clear trends (Fig. 2a–c, Fig. S1).

**Fig. 3:**
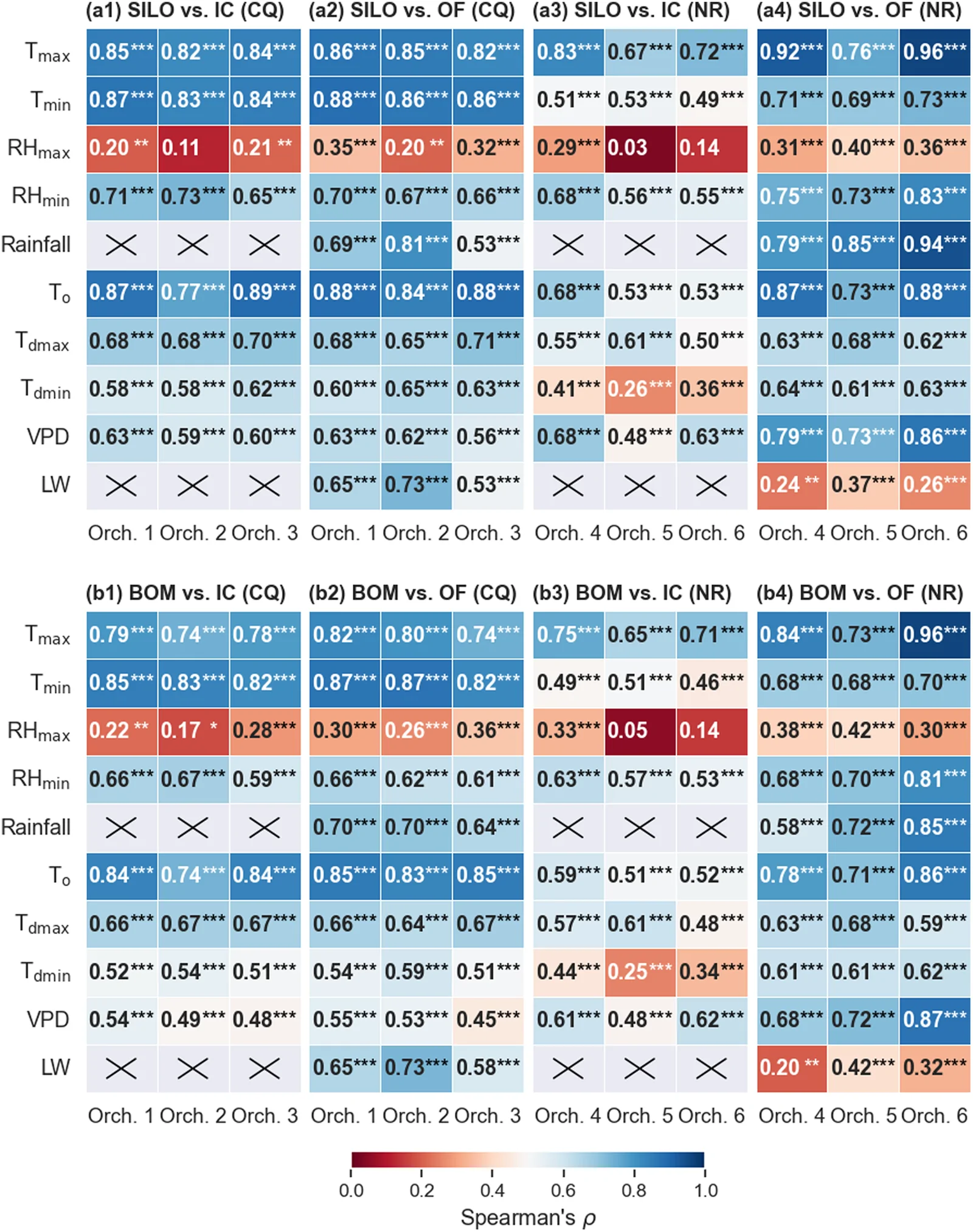
Spearman’s correlation coefficient between 10 weather variables from (a) SILO and fine-scale (in-canopy, IC; on-farm, OF) sources and (b) BOM and fine-scale (in-canopy, IC; on-farm, OF) sources across six commercial macadamia orchards in (a1–2 and b1–2) Central Queensland (Orchards 1–3) and (a3–4 and b3–4) Northern Rivers (Orchards 4–6) across three seasons (2022–2024). Statistically significant coefficients are indicated by *** (*p* < 0.001), ** (*p* < 0.01) or * (*p* < 0.05). $\:T_{dmax}$: maximum temperature; $\:T_{dmin}$: minimum temperature; $\:RH_{max}$: maximum relative humidity; $\:RH_{min}$: minimum relative humidity; $\:T_{o}$: thermal oscillation; $\:T_{dmax}$: maximum dew point temperature; $\:T_{dmin}$: minimum dew point temperature; VPD: vapour pressure deficit; LW: Leaf wetness. Crosses indicate not applicable

Correlations across all variables were generally stronger between SILO and BOM (*ρ* = 0.44–1.00, *p* < 0.001) than between in-canopy and on-farm (*ρ* = 0.40–0.97, *p* < 0.001) sources, with no correlation in $RH_{max}$ at Orchard 5 (Fig. S2). Inter-scale comparisons showed both coarse-scale sources correlated better with on-farm (*ρ* = 0.20–0.96, *p* < 0.01) than with in-canopy (*ρ* = 0.17–0.89, *p* < 0.05) sources (Fig. 3).

**Fig. 4:**
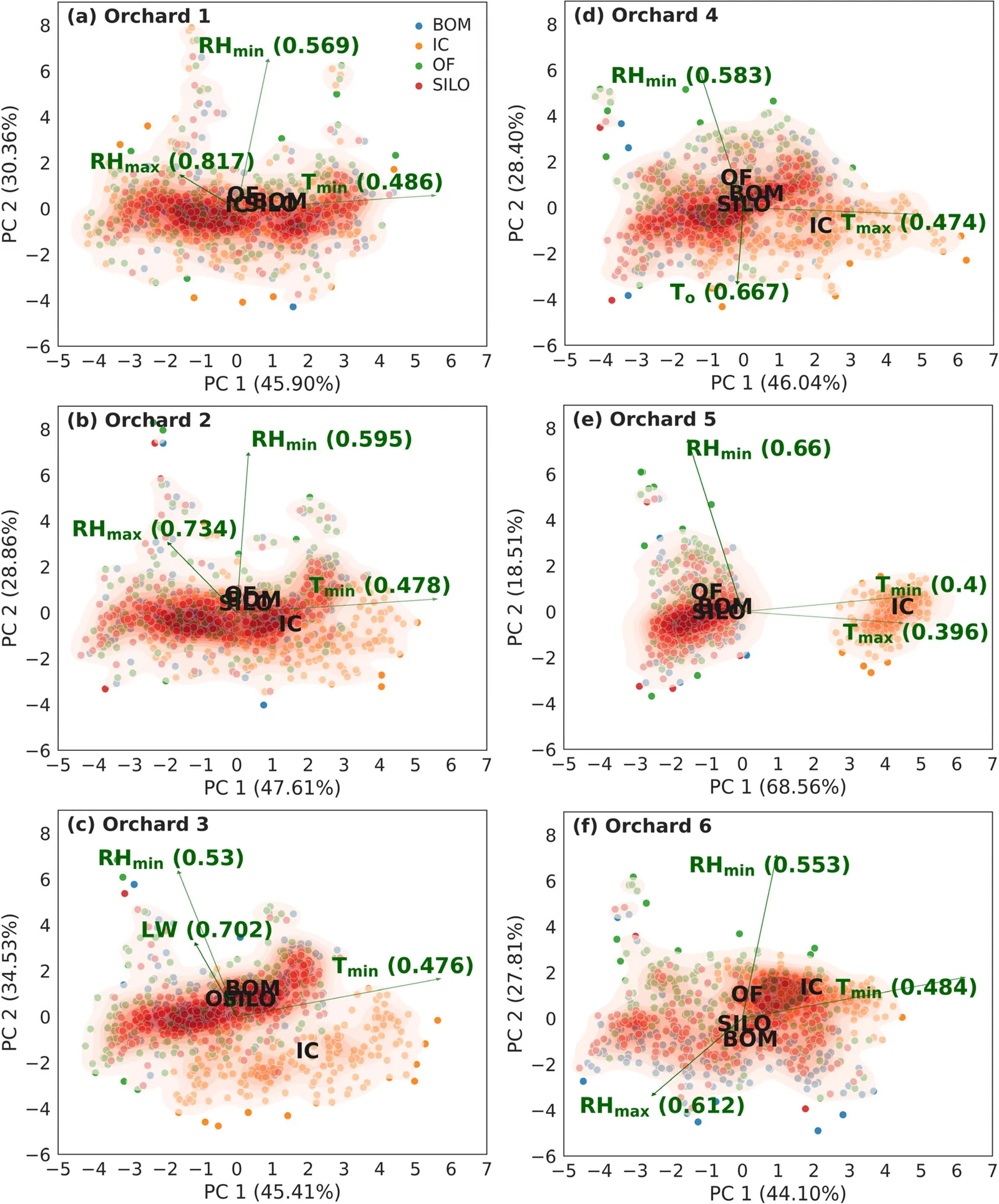
Principal component analysis (PCA) biplot of 10 weather variables from fine-scale (in-canopy, IC and on-farm, OF) and coarse-scale (SILO and BOM) sources across six commercial macadamia orchards in (a–c) Central Queensland (Orchards 1–3) and (d–f) Northern Rivers (Orchards 4–6) across three seasons (2022–2024). Variables with highest loadings are indicated in the biplot. $RH_{min}$: minimum relative humidity; $\:RH_{max}$: maximum relative humidity; $\:T_{min}$: minimum temperature; LW: leaf wetness; $\:T_{max}$: maximum temperature; $\:T_{o}$: thermal oscillation; PC: principal component

$T_{min}$ (0.40–0.49), $T_{max}$ (0.47) and $RH_{min}$ (0.53–0.66) significantly contributed to 71.92–87.07% of the total variance, as captured by principal component (PC) 1 (44.10–68.56%) and PC2 (18.51–34.53%) (Fig. 4). $T_{min}$ and $T_{max}$ were primarily associated with in-canopy (Fig. 4b–c, e–f), whereas $RH_{min}$ was primarily associated with on-farm (Fig. 4a–b, d–f). The PC3 explained additional 11.33–18.66% of the variance, with the highest contributions from $RH_{max}$ (0.61–0.82), LW (0.70) and $T_{o}$ (0.67), primarily associated with SILO and BOM (Fig. 4b–d, f). Fine-scale sources commonly showed mixed clustering, with on-farm sources predominating across orchards (Fig. S3a–b, d–f). Notably, distinct clustering was observed in Orchards 5 and 6 (Fig. S3e–f, Table 2), where data from in-canopy sources separated completely (100%) from other sources across all variables (precision = 1.00, recall = 1.00, F1-score = 1.00, overall accuracy = 0.50–0.56).

**Table 2:** Clustering performance metrics for fine-scale (in-canopy and on-farm) and coarse-scale (SILO and BOM) sources of weather data for six commercial macadamia orchards in two distinct agroecological regions, Central Queensland (Orchards 1–3) and Northern Rivers (Orchards 4–6) in 2022 to 2024 seasons

Metric	Weather data source cluster	Central Queensland			Northern Rivers		
		Orchard 1	Orchard 2	Orchard 3	Orchard 4	Orchard 5	Orchard 6
Precision^a^	In-canopy	0.24	0.80	0.00	0.12	1.00	1.00
	On-farm	0.31	0.43	0.37	0.01	0.31	0.54
	SILO	0.23	0.31	0.06	0.37	0.23	0.42
	BOM	0.22	0.32	0.29	0.37	0.48	0.37
Recall^b^	In-canopy	0.29	0.97	0.00	0.08	1.00	1.00
	On-farm	0.07	0.12	0.10	0.01	0.22	0.23
	SILO	0.21	0.34	0.07	0.53	0.28	0.52
	BOM	0.36	0.44	0.39	0.43	0.52	0.49
F1-score^c^	In-canopy	0.26	0.88	0.00	0.09	1.00	1.00
	On-farm	0.11	0.19	0.15	0.01	0.25	0.32
	SILO	0.22	0.32	0.07	0.43	0.25	0.46
	BOM	0.27	0.37	0.33	0.40	0.50	0.42
Accuracy^d^		0.23	0.47	0.14	0.26	0.50	0.56

^a^Precision is the proportion of correctly assigned sources within a cluster relative to the total number of sources assigned to that cluster

^b^Recall is the proportion of correctly assigned sources within a cluster relative to the total number of true sources belonging to that cluster

^c^F1-score is the harmonic mean of precision and recall

^d^Accuracy is the proportion of correctly classified sources across all clusters

Higher values (range = 0–1) indicate greater differences in orchard microclimate conditions captured by a source relative to the other sources of each orchard

### Predictive performance of weather variables based on sources

**Fig. 5:**
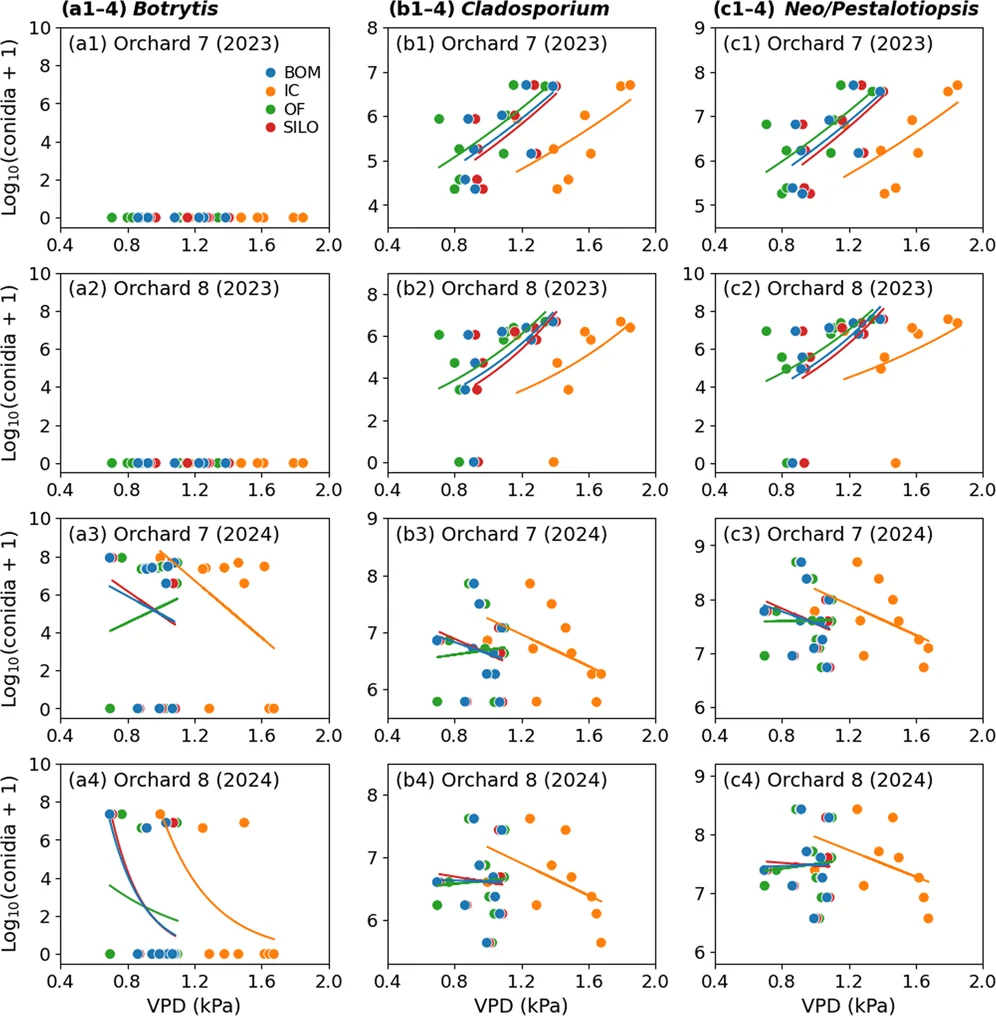
Regression of airborne conidial abundance for (a1–4) *Botrytis*, (b1–4) *Cladosporium* and (c1–4) *Neopestalotiopsis*/*Pestalotiopsis* against vapour pressure deficit (VPD) from in-canopy (IC, yellow), on-farm (OF, green), SILO (red) and BOM (blue) sources of weather data for two commercial macadamia orchards in Central Queensland (Orchards 7 and 8) during two production seasons (2023 and 2024). Solid lines and points represent the fitted regression and observed data, respectively. VPD values represent biweekly mean corresponding to the spore trapping periods

**Fig. 6:**
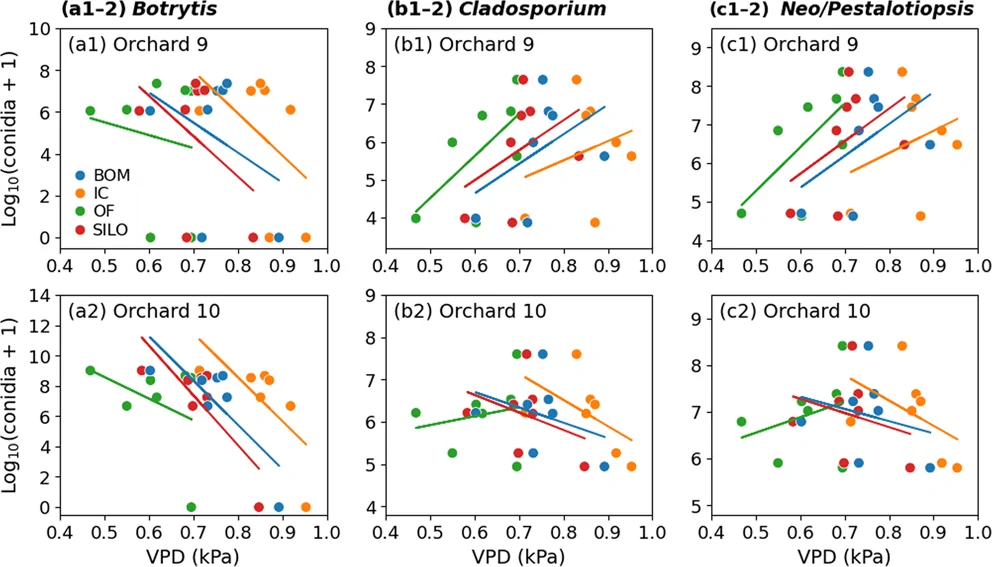
Regression of airborne conidial abundance for (a1–2) *Botrytis*, (b1–2) *Cladosporium* and (c1–2) *Neopestalotiopsis*/*Pestalotiopsis* against vapour pressure deficit (VPD) from in-canopy (IC, yellow), on-farm (OF, green), SILO (red) and BOM (blue) sources of weather data for two commercial macadamia orchards in Northern Rivers (Orchards 9 and 10) during the 2024 production season. No conidia data was collected during the 2023 season. Solid lines and points represent the fitted regression and observed data, respectively. VPD values represent biweekly mean corresponding to the spore trapping periods

Predictive models were fitted for each weather source in the four selected orchards (Figs. 5 and 6). In the 2023 season, *Botrytis* conidia were not detected and were excluded from further analyses, whereas there were rapid increases in conidia dynamics in two Orchards (7 and 8) for *Cladosporium* (Fig. 5b1 and 2) and *Neopestalotiopsis*/*Pestalotiopsis* (Fig. 5c1 and 2). The relationships were best described by exponential models (Table 3). In the 2024 season, linear models best described the gradual accumulation of the fungal conidia in all orchards, except for *Botrytis* in Orchard 8, which followed an exponential trend (Fig. 5a4).

**Table 3:** Regression equations of $\log_{10}$(number of conidia + 1) (*y*) against vapour pressure deficit (VPD) (*x*) from fine-scale (in-canopy and on-farm) and coarse-scale (SILO and BOM) sources of weather data across four commercial macadamia orchards in two agroecologial regions during the 2023 and 2024 seasons

Season	Region	Orchard label	Source of weather data	Fungal genus			Regression type
				Botrytis	Cladosporium	Neopestalotiopsis/Pestalotiopsis	
2023	Central Queensalnd	Orchard 7	In-canopy	NA	$\:y={2.861}^{0.432x}$	$\:y={3.587}^{0.385x}$	Exponential
			On-farm	NA	$\:y={3.425}^{0.492x}$	$\:y={4.225}^{0.434x}$	
			SILO	NA	$\:y={3.067}^{0.535x}$	$\:y={3.791}^{0.481x}$	
			BOM	NA	$\:y={3.242}^{0.506x}$	$\:y={3.977}^{0.457x}$	
		Orchard 8	In-canopy	NA	$\:y={0.997}^{1.022x}$	$\:y={1.920}^{0.710x}$	Exponential
			On-farm	NA	$\:y={1.589}^{1.120x}$	$\:y={2.155}^{0.983x}$	
			SILO	NA	$\:y={1.038}^{1.371x}$	$\:y={1.441}^{1.228x}$	
			BOM	NA	$\:y={1.279}^{1.237x}$	$\:y={1.632}^{1.165x}$	
2024	Central Queensalnd	Orchard 7	In-canopy	* y=-7.509x+15.745*	* y=-1.390x+8.632*	* y=-1.417x+9.601*	Linear
			On-farm	* y=4.203x+1.151*	* y=0.418x+6.276*	* y=0.073x+7.537*	
			SILO	* y=-6.154x+11.095*	* y=-1.305x+7.931*	* y=-1.367x+8.922*	
			BOM	* y=-4.795x+9.747*	* y=-0.997x+7.627*	* y=-1.078x+8.635*	
		Orchard 8	In-canopy	$\:y={209.675}^{-3.340x}$	* y=-1.284x+8.445*	* y=-1.132x+9.087*	Exponential and linear
			On-farm	$\:y={12.477}^{-1.788x}$	* y=0.264x+6.365*	* y=0.386x+7.107*	
			SILO	$\:y={314.676}^{-5.348x}$	* y=-0.441x+7.044*	* y=-0.223x+7.691*	
			BOM	$\:y={227.597}^{-5.008x}$	* y=-0.085x+6.699*	* y=0.107x+7.372*	
	Northern Rivers	Orchard 9	In-canopy	* y=-20.114x+22.025*	* y=5.080x+1.449*	* y=5.748x+1.667*	Linear
			On-farm	* y=-6.152x+8.581*	* y=11.097x-1.031*	* y=11.375x-0.411*	
			SILO	* y=-19.288x+18.344*	* y=7.918x+0.238*	* y=8.457x+0.650*	
			BOM	* y=-14.320x+15.513*	* y=7.809x-0.045*	* y=8.273x+0.398*	
		Orchard 10	In-canopy	* y=-28.911x+31.696*	* y=-6.214x+11.485*	* y=-5.397x+11.558*	Linear
			On-farm	* y=-14.238x+15.694*	* y=2.091x+4.875*	* y=3.292x+4.907*	
			SILO	* y=-32.820x+30.345*	* y=-4.150x+9.123*	* y=-3.034x+9.099*	
			BOM	* y=-29.213x+28.797*	* y=-3.632x+8.881*	* y=-2.610x+8.887*	

No conidia data was collected for Orchards 9 and 10 during the 2023 season. *NA* not applicable

**Fig. 7:**
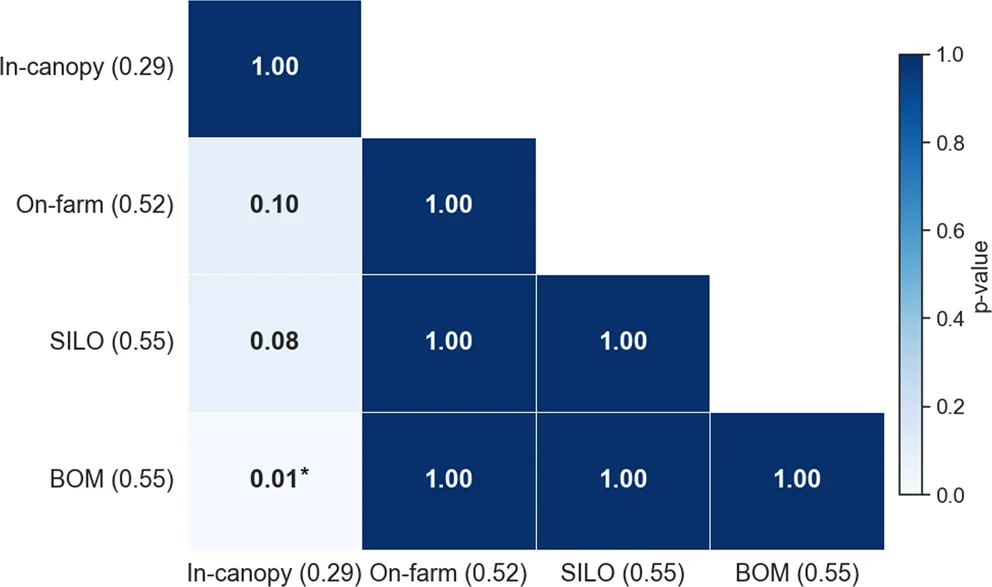
Probability (*p*) values from Dunn’s post hoc comparisons of predictive metrics for VPD-based models derived from fine-scale (in-canopy and on-farm) and coarse-scale (SILO and BOM) sources of weather data for flower blight conidia across four commercial macadamia orchards during two production seasons (2023 and 2024). Values in brackets indicate predictive robustness index (PRI) for each weather source. Statistically significant difference is indicated by *p* < 0.05 (*)

Model directionality varied among the weather sources, particularly for *Cladosporium* and *Neopestalotiopsis*/*Pestalotiopsis* in Orchards 7, 8 and 10 in the 2024 season, where on-farm-based models (Table 3) showed positive relationships, whereas in-canopy, SILO and BOM-based models showed negative relationships (Fig. 5b3-4, 5c3-4, 6b2 and 6c2), which may reflect differences in microclimatic humidity dynamics among weather sources. Subsequently, predictive performance differed significantly across pathogens, orchards and seasons (*H*(3) = 11.09, *p* = 0.01), with agreement, bias and prediction errors for airborne conidia showing wide ranges (Fig. S4). The PRI rankings of the weather sources were consistent across the tested $\:\lambda\:$ values (Table S3). Therefore, PRI values for $\:\lambda\:$ = 0.1, which applied the minimal penalty, were used to compare the robustness of weather source predictions. BOM-based models (PRI = 0.55) outperformed the in-canopy models (PRI = 0.29; *p* < 0.05) but were similar to on-farm (PRI = 0.52) and SILO (PRI = 0.55) models (*p* > 0.05; Fig. 7). In-canopy, on-farm and SILO models showed comparable predictive performance (*p* > 0.05).

## Discussion

This study identified variability in weather variables from fine (in-canopy and on-farm) and coarse (SILO and BOM) spatial scale sources and their accuracy in predicting flower blight fungal conidia dynamics. Inter-scale correlations ranged from weak to strong, and differences among sources were mainly associated with $T_{min}$ and $RH_{min}$. In-canopy sources showed more temporal stability and formed distinct clusters in 33% of orchards, whereas SILO and on-farm were more variable and often clustered with BOM sources. These source-specific characteristics was associated with differences in predictive performance, with on-farm, SILO and BOM sources outperforming in-canopy sources in predicting *Botrytis*,* Cladosporium* and *Neopestalotiopsis*/*Pestalotiopsis* conidia dynamics.

Weather-based prediction models are crucial for effective disease management (Fenu and Malloci 2021; Jensen et al. 2025). However, variations in microclimate due to different weather sources can be associated with the accuracy of these predictions (El Jarroudi et al. 2017; Rodríguez-Moreno et al. 2020). This study identified a more stable microclimate using in-canopy sources compared with on-farm, SILO and BOM sources, primarily characterised by reduced temporal variability in $RH_{min}$, $RH_{max}$ and VPD. This stability possibly arises from a buffering effect of the canopy that moderates atmospheric fluctuations (Murakami et al. 2022; Spronken-Smith and Oke 1998). Depending on conditions such as cloud cover and sunlight, canopy temperatures can be 2 °C to 5 °C cooler and relative humidity 15% to 25% higher than ambient conditions outside the canopy (van Westreenen et al. 2020).

Variables from SILO and BOM were strongly correlated across orchards despite being 9 km to 35 km apart, reflecting SILO’s derivation from BOM point-based and gridded (0.05° × 0.05°) datasets (Zaman et al. 2024). Rainfall remained relatively source-specific and consistent with previous studies (Rivera et al. 2018; Shah et al. 2019). This is likely due to differences in local topography and precipitation events, as rainfall is highly location-specific and may not be fully captured by grids (El Jarroudi et al. 2017; Schleiss and Smith 2016). Conversely, in-canopy and on-farm sources, though located within the same orchard, showed lower correlations with better alignment in CQ than in NR. These differences may be associated with canopy density and light interception gradients (Schreiber et al. 2025; van Westreenen et al. 2020), where trees in NR orchards were denser, older canopies (> 8 m inter-row, > 12 years) whereas CQ orchards were younger and sparser (< 7 m inter-row, < 12 years). $T_{max}$ and LW showed the strongest and weakest spatial correlations among sources, respectively, consistent with previous studies (Ashraf et al. 1997; Gleason et al. 2008). It is important to note that spatial distance had a minimal effect on correlations between fine-scale and coarse-scale weather sources. These findings suggest that local microclimatic conditions may play a greater role than spatial distance alone in explaining the observed associations.

$T_{min}$ and $RH_{min}$ were the primary contributors to variation among the sources, reflecting differences in microclimate, with in-canopy sources aligned with $T_{min}$ and on-farm and coarse-scale sources aligned with $RH_{min}$. This contrast has been demonstrated in previous studies (Ashraf et al. 1997; Murakami et al. 2022), and likely reflects canopy depth effects (De Frenne et al. 2019). In-canopy RH varies substantially across canopies, with temperature dominating RH variations in the upper canopy and water vapour pressure influencing RH in the lower canopy (van Westreenen et al. 2020). Consequently, $T_{min}$ is maintained primarily within the canopy (Schreiber et al. 2025), consistent with its stronger correlation with in-canopy sources. Clearer differences among the sources were observed from the clustering analysis, consistent with earlier research (Sönmez and Kömüşcü 2011; Yeo et al. 2020). In-canopy sources consistently formed fully or near-distinct clusters (> 97% agreement) in 50% of orchards, highlighting their distinctive spatial variability in microclimatic patterns, in contrast to BOM, which were more representative of overall orchard-level microclimate. Data from SILO and on-farm often clustered with BOM or in-canopy sources, reflecting their greater similarity in microclimates.

While on-farm and coarse-scale data often provide a reasonable approximation of orchard-level conditions, the distinctive orchard specific microclimates captured by in-canopy sources may be associated with pathogen inoculum dynamics and host colonisation rates, making them valuable for disease prediction (de Mol et al. 2018; Pangga et al. 2011). However, the contrasting VPD-conidia relationship directions observed between on-farm, and in-canopy and regional weather sources in some orchards may reflect differences in spatial representation of orchard-specific microclimatic conditions among weather sources. Exploratory analysis of VPD component variables suggested that more persistent $RH_{max}$ conditions were captured by on-farm weather source, which showed lower $RH_{max}$ variability compared with in-canopy, SILO and BOM weather sources. Differences in $RH_{max}$ variability among the weather sources may have contributed to the contrasting VPD-conidia relationship directions between on-farm, and in-canopy, SILO and BOM weather sources, possibly due to the strong effect of moisture availability on conidial abundance (De Cól et al. 2024; Mata et al. 2026; Meno et al. 2022).

Conversely, coarse-scale weather data provided more consistent and generalisable predictions for flower blight fungal conidia, whereas fine-scale data exhibited more orchard-specific and variable predictive performance across orchards. This may suggest a trade-off between local microclimatic specificity and model generalisability. Coarse-scale weather sources may smooth orchard-specific microclimatic fluctuations and provide a more stable representation of weather conditions across orchards, which may contribute to more consistent predictive performance (De Cól et al. 2024). In contrast, fine-scale weather sources may capture localised weather variability that is biologically relevant within individual orchards but inconsistent across orchards, thereby increasing predictive variability and reducing model generalisability (Wang et al. 2000). The higher predictive performance of on-farm sources than in-canopy sources may be associated with canopy structural characteristics (Tivoli et al. 2013). However, attributes such as inter-row canopy diameter ($\:{R}^{2}$ = 0.19) and tree age ($\:{R}^{2}$ = 0.18) were only weakly associated with in-canopy predictive accuracy, indicating that structural attributes alone may not adequately explain the predictive performance of in-canopy weather data (Chang et al. 2007).

The relatively higher and more stable VPD conditions (mean = 1.29 kPa) observed within canopies were likely suboptimal for conidia development by the pathogens (O’neill et al. 1997; Prasannath et al. 2022a), contributing to the reduced conidia predictive performance of in-canopy weather data. However, the mean VPD of on-farm (0.87 kPa), SILO (0.94 kPa) and BOM (0.93 kPa) sources aligns closely with the VPD range for conidial development of all flower blight pathogens, potentially accounting for their higher and more consistent predictive performance relative to the in-canopy source. The relatively larger VPD range observed for the in-canopy source (1.13 kPa) compared with the on-farm (0.87 kPa), SILO (0.83 kPa) and BOM (0.78 kPa) sources may also underlie the greater inconsistency in the predictive performance of in-canopy VPD across orchards. These observed in-canopy VPD characteristics suggest that additional factors may be associated with microclimate beyond canopy diameter ($\:{R}^{2}$ = 0.04) and age ($\:{R}^{2}$ = 0.07). Cultivar characteristics such as leaf area index, together with sensor-related factors including height and cardinal orientation, are associated with vapour pressure dynamics (Murakami et al. 2022; Schreiber et al. 2025; van Westreenen et al. 2020). It should be noted that other weather variables from in-canopy sources, aside from VPD, may provide better predictions of flower blight fungal conidia, as demonstrated in previous study (Rodríguez-Moreno et al. 2020).

The comparable predictive performance between in-canopy, on-farm and SILO (< 4 km apart), in contrast to BOM (> 13 km apart), suggests that in-canopy and open-air microclimates share partial spatial similarity, consistent with Tobler (1970). Overall, these findings emphasise that the choice of weather data source is strongly associated with model performance, often more than variable selection or analytical methods, as highlighted by previous studies (Jensen et al. 2025; Shah et al. 2019). Accordingly, users of weather-driven disease models should carefully consider input data sources to ensure robust management decisions. Weather data from on-farm and coarse-scale sources may be more suitable for predictive models intended for broader applicability across sites, whereas models based on in-canopy weather data may be more appropriate for site-specific applications due to the highly localised nature of in-canopy microclimates.

Limited historical data from fine-scale stations restricted the study to 10 orchards with complete two- to three-season records. While the study demonstrated the impact of variability among weather data sources on disease prediction accuracy, this was limited to flower blight pathogens and univariate VPD-based regression models. Although flower blight dynamics are associated with multiple interacting environmental variables, the univariate modelling approach may not fully capture the complexity of disease dynamics. Additionally, LOO-CV does not preserve temporal ordering and may provide optimistic estimates when applied to time-dependent data. Therefore, future forecasting studies should consider blocked or rolling-origin validation procedures.

## Conclusion

This study evaluated fine (in-canopy and on-farm) and coarse (regional: SILO and BOM) spatial scale weather sources across two major crop production regions in Australia to assess their reliability for weather-based disease prediction. Weather variables differed considerably, with the variation driven more strongly by data source than by spatial scale. The contrasting relationship directions among weather sources are particularly interesting and potentially represent one of the most novel findings of this study. Predictive performance of models for conidial abundance of the fungal pathogens *Botrytis*, *Cladosporium* and *Neopestalotiopsis*/*Pestalotiopsis* varied markedly among weather sources, with regional sources generally providing the most reliable input for disease forecasting. These findings provide a practical framework for improving weather-based disease management across cropping systems.

## Supplementary Information

Below is the link to the electronic supplementary material.

Supplementary Material 1 (DOCX 702 KB)

## Data Availability

All the datasets and code used for the analyses in this study are available in The University of Queensland Research Data Manager (UQRDM). Further inquiries can be directed to the corresponding authors.
