# Supplementary material for: Weather data sources influence predictive performance of disease forecasting models
Source: Int J Biometeorol. 2026 Aug 18;70(9):238. doi: 10.1007/s00484-026-03285-3 (PMC13486128; doi:10.1007/s00484-026-03285-3)
Supplement: Supplementary file 1 [file 484_2026_3285_MOESM1_ESM.docx]

**Weather data sources influence predictive performance of disease forecasting models**

International Journal of Biometeorology

Theophilus Abonyi Mensah*^1^, Neil White^1,2^, Vivian Rincón‐Flórez^1^, Olufemi A. Akinsanmi*^1^

^1^Centre for Horticultural Science, Queensland Alliance for Agriculture and Food Innovation, The University of Queensland, Ecosciences Precinct, Brisbane, QLD 4102, Australia

^2^Queensland Department of Primary Industries, Toowoomba, QLD 4350, Australia

*Correspondence

E-mail addresses: t.mensah@uq.net.au (T. A. Mensah) and o.akinsanmi@uq.edu.au (O. A. Akinsanmi)

**Table S1** Lag-1 serial correlation ($r_{1}$) coefficients for 10 weather variables from four weather sources across six commercial macadamia orchards in Central Queensland (Orchards 1–3) and Northern Rivers (Orchards 4–6) across three production seasons (2022–2024).

| Orchard | Weather source | $T_{\max}$ | $T_{\min}$ | $\mathrm{RH}_{\max}$ | $\mathrm{RH}_{\min}$ | Rainfall | $T_{o}$ | $T_{\mathrm{dmin}}$ | $T_{\mathrm{dmax}}$ | VPD | LW |
| --- | --- | --- | --- | --- | --- | --- | --- | --- | --- | --- | --- |
| Orchard 1 | In-canopy | 0.90 | 0.90 | 0.38 | 0.58 | NA | 0.60 | 0.90 | 0.81 | 0.72 | 0.51 |
|  | On-farm | 0.89 | 0.90 | 0.34 | 0.56 | 0.15 | 0.63 | 0.91 | 0.81 | 0.67 | 0.46 |
|  | SILO | 0.94 | 0.94 | 0.26 | 0.60 | 0.23 | 0.68 | 0.91 | 0.85 | 0.80 | 0.23 |
|  | BOM | 0.93 | 0.94 | 0.18 | 0.56 | 0.13^†^ | 0.67 | 0.89 | 0.82 | 0.71 | 0.23 |
|  |  |  |  |  |  |  |  |  |  |  |  |
| Orchard 2 | In-canopy | 0.95 | 0.96 | 0.59 | 0.67 | NA | 0.59 | 0.93 | 0.88 | 0.88 | NA |
|  | On-farm | 0.93 | 0.93 | 0.38 | 0.64 | 0.12 | 0.62 | 0.93 | 0.87 | 0.73 | 0.31 |
|  | SILO | 0.94 | 0.94 | 0.26 | 0.60 | 0.23 | 0.68 | 0.91 | 0.85 | 0.80 | 0.23 |
|  | BOM | 0.93 | 0.94 | 0.18 | 0.56 | 0.13^†^ | 0.67 | 0.89 | 0.82 | 0.71 | 0.23 |
|  |  |  |  |  |  |  |  |  |  |  |  |
| Orchard 3 | In-canopy | 0.92 | 0.94 | 0.54 | 0.67 | NA | 0.65 | 0.93 | 0.88 | 0.87 | NA |
|  | On-farm | 0.91 | 0.92 | 0.31 | 0.68 | 0.20 | 0.65 | 0.93 | 0.87 | 0.74 | 0.30 |
|  | SILO | 0.94 | 0.95 | 0.28 | 0.61 | 0.47 | 0.68 | 0.91 | 0.86 | 0.81 | 0.23 |
|  | BOM | 0.93 | 0.94 | 0.18 | 0.56 | 0.13^†^ | 0.67 | 0.89 | 0.82 | 0.71 | 0.23 |
|  |  |  |  |  |  |  |  |  |  |  |  |
| Orchard 4 | In-canopy | 0.87 | 0.97 | 0.61 | 0.44 | NA | 0.37 | 0.94 | 0.89 | 0.80 | NA |
|  | On-farm | 0.83 | 0.92 | 0.51 | 0.49 | 0.29 | 0.31 | 0.92 | 0.87 | 0.50 | 0.32 |
|  | SILO | 0.88 | 0.93 | 0.22 | 0.49 | 0.38 | 0.48 | 0.91 | 0.89 | 0.59 | 0.22 |
|  | BOM | 0.86 | 0.92 | 0.28 | 0.49 | 0.34 | 0.44 | 0.90 | 0.88 | 0.50 | 0.08^†^ |
|  |  |  |  |  |  |  |  |  |  |  |  |
| Orchard 5 | In-canopy | 0.77 | 0.92 | 0.25 | 0.73 | NA | 0.63 | 0.80 | 0.87 | 0.38 | NA |
|  | On-farm | 0.82 | 0.92 | 0.46 | 0.55 | 0.33 | 0.48 | 0.92 | 0.87 | 0.50 | 0.37 |
|  | SILO | 0.87 | 0.92 | 0.26 | 0.49 | 0.39 | 0.46 | 0.91 | 0.89 | 0.52 | 0.19 |
|  | BOM | 0.86 | 0.92 | 0.28 | 0.49 | 0.34 | 0.44 | 0.90 | 0.88 | 0.50 | 0.08^†^ |
|  |  |  |  |  |  |  |  |  |  |  |  |
| Orchard 6 | In-canopy | 0.85 | 0.91 | 0.87 | 0.45 | NA | 0.18 | 0.93 | 0.81 | 0.59 | NA |
|  | On-farm | 0.83 | 0.92 | 0.45 | 0.45 | 0.43 | 0.31 | 0.92 | 0.87 | 0.51 | 0.42 |
|  | SILO | 0.87 | 0.93 | 0.21 | 0.49 | 0.41 | 0.47 | 0.91 | 0.89 | 0.61 | 0.25 |
|  | BOM | 0.86 | 0.93 | 0.23 | 0.51 | 0.40 | 0.48 | 0.91 | 0.89 | 0.64 | 0.20 |

NA: not applicable; $T_{\max}$: maximum temperature; $T_{\min}$: minimum temperature; $\mathrm{RH}_{\max}$: maximum relative humidity; $\mathrm{RH}_{\min}$: minimum relative humidity; $T_{o}$: thermal oscillation; $T_{\mathrm{dmax}}$: maximum dew point temperature; $T_{\mathrm{dmin}}$: minimum dew point temperature; VPD: vapour pressure deficit; LW: leaf wetness; BOM: Bureau of Meteorology; SILO: Scientific Information for Land Owners. Coefficients were significant at *p* < 0.05, except values indicated by ^†^.

**Table S2** Thiel-Sen’s slope (× ${10}^{-2}$) for trends in nine weather variables from four weather sources across four developmental stages of six commercial macadamia orchards in Central Queensland (Orchards 1–3) and Northern Rivers (Orchards 4–6) across three production seasons (2022–2024).

| Variable | Weather data source | Central Queensland | | | |  | Northern Rivers | | |
| --- | --- | --- | --- | --- | --- | --- | --- | --- | --- |
|  |  |  | Orchard 1 | Orchard 2 | Orchard 3 |  | Orchard 4 | Orchard 5 | Orchard 6 |
| $T_{\max}$ | In-canopy |  | 0.55 | 0.36 | 0.48 |  | 1.01 | 0.63 | 0.52 |
|  | On-farm |  | 0.57 | 0.39 | 0.52 |  | 0.98 | 0.75 | 0.95 |
|  | SILO |  | 0.25 | 0.25 | 0.31 |  | 0.68 | 0.68 | 0.77 |
|  | BOM |  | 0.29 | 0.29 | 0.29 |  | 0.66 | 0.66 | 0.84 |
|  |  |  |  |  |  |  |  |  |  |
| $T_{\min}$ | In-canopy |  | 0.72 | 0.29 | 0.56 |  | 0.21 | 0.44 | 0.40 |
|  | On-farm |  | 0.68 | 0.47 | 0.75 |  | 0.43 | 0.50 | 0.49 |
|  | SILO |  | 0.40 | 0.40 | 0.41 |  | 0.51 | 0.52 | 0.54 |
|  | BOM |  | 0.42 | 0.42 | 0.42 |  | 0.50 | 0.50 | 0.61 |
|  |  |  |  |  |  |  |  |  |  |
| $\mathrm{RH}_{\max}$ | In-canopy |  | –0.47 | –0.32 | –0.34 |  | 0.12 | 2.71 | 1.16 |
|  | On-farm |  | –0.09 | 0.39 | –0.34 |  | 1.25 | 0.75 | 0.71 |
|  | SILO |  | –2.06 | –2.06 | –1.80 |  | 0.00 | 0.16 | –0.01 |
|  | BOM |  | –2.33 | –2.33 | –2.33 |  | 0.42 | 0.42 | –0.19 |
|  |  |  |  |  |  |  |  |  |  |
| $\mathrm{RH}_{\min}$ | In-canopy |  | –0.29 | –0.21 | 0.70 |  | –0.25 | 1.90 | 1.59 |
|  | On-farm |  | 0.77 | 1.27 | 1.22 |  | 1.23 | 2.04 | 0.73 |
|  | SILO |  | 0.32 | 0.32 | 0.46 |  | 0.99 | 1.49 | 0.67 |
|  | BOM |  | 0.70 | 0.70 | 0.70 |  | 1.80 | 1.80 | 0.59 |
|  |  |  |  |  |  |  |  |  |  |
| Rainfall | On-farm |  | 0.00 | 0.01 | 0.03 |  | 0.23 | 0.17 | 0.04 |
|  | SILO |  | 0.11 | 0.11 | 0.09 |  | 0.23 | 0.20 | 0.05 |
|  | BOM |  | 0.01 | 0.01 | 0.01 |  | 0.05 | 0.05 | 0.01 |
|  |  |  |  |  |  |  |  |  |  |
| $T_{o}$ | In-canopy |  | –0.36 | 0.03 | –0.73 |  | 0.87 | –0.65 | –0.14 |
|  | On-farm |  | –0.71 | –0.46 | –0.83 |  | 0.29 | –0.68 | 0.28 |
|  | SILO |  | –0.61 | –0.61 | –0.64 |  | –0.46 | –0.50 | –0.41 |
|  | BOM |  | –0.62 | –0.62 | –0.62 |  | –0.52 | –0.52 | –0.54 |
|  |  |  |  |  |  |  |  |  |  |
| $T_{\mathrm{dmax}}$ | In-canopy |  | 1.07 | 0.63 | 0.85 |  | 0.84 | 0.74 | 0.73 |
|  | On-farm |  | 1.12 | 0.86 | 0.90 |  | 0.82 | 0.77 | 0.89 |
|  | SILO |  | 0.73 | 0.73 | 0.77 |  | 0.68 | 0.76 | 0.71 |
|  | BOM |  | 0.90 | 0.90 | 0.90 |  | 0.78 | 0.78 | 0.70 |
|  |  |  |  |  |  |  |  |  |  |
| $T_{\mathrm{dmin}}$ | In-canopy |  | 0.68 | 0.41 | 0.57 |  | 0.40 | 1.11 | 0.42 |
|  | On-farm |  | 0.66 | 0.46 | 0.64 |  | 0.51 | 0.51 | 0.51 |
|  | SILO |  | 0.57 | 0.57 | 0.56 |  | 0.60 | 0.66 | 0.68 |
|  | BOM |  | 0.66 | 0.66 | 0.66 |  | 0.70 | 0.70 | 0.69 |
|  |  |  |  |  |  |  |  |  |  |
| VPD | In-canopy |  | 0.09 | 0.06 | 0.08 |  | 0.10 | 0.09 | 0.02 |
|  | On-farm |  | 0.06 | 0.06 | 0.07 |  | 0.07 | 0.04 | 0.09 |
|  | SILO |  | 0.06 | 0.06 | 0.07 |  | 0.08 | 0.08 | 0.09 |
|  | BOM |  | 0.08 | 0.08 | 0.08 |  | 0.07 | 0.07 | 0.10 |

$T_{\max}$: maximum temperature; $T_{\min}$: minimum temperature; $\mathrm{RH}_{\max}$: maximum relative humidity; $\mathrm{RH}_{\min}$: minimum relative humidity; $T_{o}$: thermal oscillation; $T_{\mathrm{dmax}}$: maximum dew point temperature; $T_{\mathrm{dmin}}$: minimum dew point temperature; VPD: vapour pressure deficit; BOM: Bureau of Meteorology; SILO: Scientific Information for Land Owners. Leaf wetness was not applicable for the analysis.

**Fig. S1** Trends in nine weather variables from fine-scale (in-canopy and on-farm) and coarse-scale (SILO and BOM) sources across six commercial macadamia orchards in (a1) Central Queensland (Orchards 1–3) and (a2) Northern Rivers (Orchards 4–6) across three production seasons (2022–2024) based on Mann-Kendall *Z* statistic ($Z_{\mathrm{mk}}$). Positive and negative signs indicate increasing and decreasing trends, respectively. Statistically significant trends are indicated by *** (*p* < 0.001), ** (*p* < 0.01) or * (*p* < 0.05). $T_{\max}$: maximum temperature; $T_{\min}$: minimum temperature; $\mathrm{RH}_{\max}$; maximum relative humidity; $\mathrm{RH}_{\min}$: minimum relative humidity; $T_{o}$: thermal oscillation; $T_{\mathrm{dmax}}$: maximum dew point temperature; $T_{\mathrm{dmin}}$: minimum dew point temperature; VPD: vapour pressure deficit. Leaf wetness was not applicable for $Z_{\mathrm{mk}}$ analysis.

**Fig. S2** Spearman’s correlation coefficients between (a) eight weather variables from fine-scale sources (in-canopy and on-farm) and (b) 10 weather variables from coarse-scale sources (SILO and BOM) across six commercial macadamia orchards in (a1 and b1) Central Queensland (Orchards 1–3) and (a2 and b2) Northern Rivers (Orchards 4–6) across three production seasons (2022–2024). Statistically significant correlations are indicated by *** (*p* < 0.001). $T_{\max}$: maximum temperature; $T_{\min}$: minimum temperature; $\mathrm{RH}_{\max}$: maximum relative humidity; $\mathrm{RH}_{\min}$: minimum relative humidity; $T_{o}$: thermal oscillation; $T_{\mathrm{dmax}}$: maximum dew point temperature; $T_{\mathrm{dmin}}$: minimum dew point temperature; VPD: vapour pressure deficit; LW: leaf wetness. LW and rain were not applicable for fine-scale sources.

**Fig. S3** Percentage association of 10 weather variables from in-canopy, on-farm, SILO and BOM sources with each source across six commercial macadamia orchards in (a–c) Central Queensland (Orchards 1–3) and (d–f) Northern Rivers (Orchards 4–6) across three production seasons (2022–2024), as shown in the confusion matrices. Diagonal values represent the proportion of weather conditions uniquely captured by each source, while off-diagonal values indicate similarity in weather conditions captured between sources.

**Fig. S4** Predictive metrics from cross-validation of VPD-based models using in-canopy (IC), on-farm (OF), SILO and BOM source data for conidial abundance of (a1–6) *Botrytis*, (b1–6) *Cladosporium* and (c1–6) *Neopestalotiopsis*/*Pestalotiopsis* in four commercial macadamia orchards in Central Queensland (CQ; Orchards 7 and 8) and Northern Rivers (NR; Orchards 9 and 10) during two production seasons (2023 and 2024). No conidia data were collected for Orchards 9 and 10 during the 2023 season. LCCC: Lin’s concordance correlation coefficient (best = 1); CRM: Coefficient of residual mass (best = 0); NRMSE: Normalised root mean squared error (best = 0). Crosses indicate not applicable.

**Table S3** Prediction robustness index of four weather sources across weighting parameter ($\lambda$) values ranging from 0 to 1.

| $\lambda$ | In-canopy  (0.34, 0.47) | On-farm  (0.56, 0.45) | SILO  (0.58, 0.37) | BOM  (0.59, 0.37) |
| --- | --- | --- | --- | --- |
| 0.0 | 0.34 | 0.56 | 0.58 | 0.59 |
| **0.1** | **0.29** | **0.52** | **0.55** | **0.55** |
| 0.2 | 0.25 | 0.47 | 0.51 | 0.51 |
| 0.3 | 0.20 | 0.43 | 0.47 | 0.48 |
| 0.4 | 0.15 | 0.38 | 0.44 | 0.44 |
| 0.5 | 0.11 | 0.34 | 0.40 | 0.40 |
| 0.6 | 0.06 | 0.29 | 0.36 | 0.36 |
| 0.7 | 0.01 | 0.25 | 0.33 | 0.33 |
| 0.8 | –0.03 | 0.20 | 0.29 | 0.29 |
| 0.9 | –0.08 | 0.16 | 0.25 | 0.25 |
| 1.0 | –0.13 | 0.11 | 0.21 | 0.21 |

Values in bracket indicate $\overline{N}$, ${SD}_{n}$ of each weather source.

The $\lambda$ value in bold was selected.
